# Blood Transfusion Utilization in Patients with Severe Coronavirus Disease 2019 in the Republic of Korea: A Nationwide Population-Based Study

**DOI:** 10.3390/jcm13237327

**Published:** 2024-12-02

**Authors:** Young Joo Oh, Jeong Yeon Kim, Jin Woong Suh, Yujin Jeong, Yumin Choi, Hyung-Jun Lim, Jang Wook Sohn, Ki Hoon Ahn, Young Kyung Yoon

**Affiliations:** 1 Division of Infectious Diseases, Department of Internal Medicine, Korea University College of Medicine, Seoul 02841, Republic of Korea; valen2337@naver.com (Y.J.O.); agisae92@gmail.com (J.Y.K.); sunthes@naver.com (J.W.S.); jwsohn@korea.ac.kr (J.W.S.); 2 Institute of Emerging Infectious Diseases, Korea University, Seoul 02841, Republic of Korea; 3 Department of Biostatistics, Korea University College of Medicine, Seoul 02841, Republic of Korea; wjddbwls1020@korea.ac.kr; 4 School of Mechanical Engineering, Korea University College of Engineering, Seoul 02841, Republic of Korea; ytooom018@naver.com; 5 Department of Bigdata Convergence, Korea University Graduate School of SW&AI Convergence, Seoul 02841, Republic of Korea; limtony1@naver.com; 6 Department of Obstetrics and Gynecology, Korea University College of Medicine, Seoul 02841, Republic of Korea; 7 Korea University Anam Hospital Bloodless Medicine Center, Seoul 02841, Republic of Korea

**Keywords:** blood transfusion, COVID-19, SARS-CoV-2, mortality

## Abstract

**Background**: The coronavirus disease 2019 (COVID-19) pandemic has significantly affected both supply and demand for blood transfusion. This study aimed to investigate the prescription patterns of blood products and identify the risk factors for blood transfusion in patients with severe COVID-19. **Methods**: This nationwide population-based cohort study was performed in the Republic of Korea between January 2021 and December 2021. Adult patients (≥19 years) with severe COVID-19 who received oxygen therapy were examined. Data were obtained from the National Health Insurance Service database. **Results**: During the study period, 41,203 patients developed severe COVID-19. The overall transfusion rate was 7.11%, with 14,477 units of packed red blood cells, 4501 units of fresh frozen plasma, 20,532 units of platelet concentrates, and 1875 units of cryoprecipitate. Blood transfusion was independently associated with 90-day mortality (hazard ratio: 1.36, 95% confidence interval: 1.21–1.53, *p* < 0.001). Multivariate logistic regression analysis identified underlying chronic kidney disease; exposure to surgery, thrombolysis, or thrombectomy; renal replacement therapy; mechanical ventilation; and extracorporeal membrane oxygenation as significant risk factors for blood transfusion in patients with severe COVID-19. Conversely, underlying cerebrovascular diseases, a Charlson Comorbidity Index score of ≥6, admission to intensive care units, and anticoagulant use were identified as protective factors. **Conclusions**: This study provides an overview of blood transfusion patterns in patients with severe COVID-19. Understanding these patterns may help optimize the stockpiling of medical resources.

## 1. Introduction

The coronavirus disease 2019 (COVID-19) pandemic, caused by the severe acute respiratory syndrome coronavirus 2 (SARS-CoV-2), has led to significant changes in global healthcare delivery. Moreover, it has posed an unprecedented challenge to blood banking and transfusion medicine [1]. Although, SARS-CoV-2, similar to other respiratory viruses, is not transmitted through blood transfusion [2], several critical issues related to blood transfusion have emerged during the pandemic. These challenges include ensuring a safe blood supply, recruiting donors, managing logistical hurdles, and addressing the growing demand for therapeutic use of COVID-19 convalescent plasma [3,4].

COVID-19 presents a broad spectrum of clinical manifestations, ranging from asymptomatic cases to critical illness. Generally, most patients with mild COVID-19 do not require blood transfusion. A previous study reported that only 0.36% of non-intensive care unit (ICU) patients with COVID-19 required packed red blood cells (pRBC), whereas 36.8% of ICU patients required pRBC transfusions, with lower demand for fresh frozen plasma (FFP) and platelet transfusion [5].

An Italian study reported that 39% of patients required transfusions, primarily due to anemia [6]. Severe organ dysfunctions, including thrombocytopenia and disseminated intravascular coagulation (DIC), may influence transfusion needs in patients with COVID-19 [7]. Coagulation disturbances, such as elevated fibrinogen and D-dimer levels, are poor prognostic markers with a higher incidence of thrombotic complications [8,9]. To mitigate thrombotic risks, many physicians have adopted expert recommendations for anticoagulation therapy in patients with severe COVID-19, although this may occasionally lead to significant bleeding [7,10]. Additionally, procedures such as extracorporeal membrane oxygenation (ECMO) in patients with severe COVID-19 may also necessitate blood component transfusions [11].

However, detailed data on blood transfusion utilization in patients with severe COVID-19 requiring oxygen therapy remain limited. Understanding the blood products required for treating severe COVID-19 is crucial for improving preparedness for future pandemics. Therefore, this population-based study aimed to investigate blood component usage and identify risk factors for blood transfusions in patients with severe COVID-19 in the Republic of Korea (ROK), as well as factors predisposing to mortality.

## 2. Materials and Methods

### 2.1. Study Design

This nationwide population-based cohort study extracted claims data from the ROK’s National Health Insurance Service-National Health Information Database (NHIS-NHID) between January 2021 and December 2021 to investigate the blood transfusion profile of patients with severe COVID-19. The claims data collected from the NHIS-NHID cover nearly all residents of the ROK, which has a population of 52 million.

Individuals aged ≥19 years diagnosed with COVID-19 based on the International Classification of Diseases, tenth revision (ICD-10), codes U07.1, U07.2, B34.2, and B97.2, were examined. Each patient was stratified using an 8-point COVID-19 ordinal scale developed by the National Institute of Allergy and Infectious Diseases [12]. Our analysis included patients with severe disease requiring oxygen therapy (≥5 points). In patients who experienced multiple episodes of COVID-19, the data from the first episode were included in the analysis to avoid bias from duplicate entries. Additionally, transfusion history within 3 months of COVID-19 diagnosis was included in the analysis.

Due to the nature of the study using population-based claim data, the decision on whether or not to transfuse blood in each patient depended on the physician’s discretion. Conversely, during the study period, the blood products were reimbursed only for patients meeting the criteria for insurance coverage from the NHIS-NHID in the ROK: 7 g/dL for RBC transfusion, 50,000/μL for platelets, international normalized ratio greater than 1.7 for plasma, and fibrinogen below 100 mg/dL for cryoprecipitate.

### 2.2. Study Outcomes

The primary objective of this study was to describe the patterns of prescribed blood products for patients with severe COVID-19. The secondary objective was to investigate the predictors of 90-day mortality. Furthermore, the tertiary outcome was the identification of risk factors associated with blood transfusion. Our findings will aid in understanding the clinical implications of blood transfusions in patients with severe COVID-19.

### 2.3. Definition and Measurement

The blood products analyzed in this study included pRBC, FFP, platelets, and cryoprecipitates. The transfusion-related ICD-10 codes were pRBC (X2021, X2022, X2031, X2032, X2091, X2092, X2111, X2112, and X2512), FFP (X2041, X2042, X2051, and X2052), platelets (X2081, X2082, X2121, X2122, X2501, X2511, and X2513), and cryoprecipitate (X2061 and X2062). The data collected included age, sex, date of treatment initiation, admission to ICUs, comorbidities, primary discharge diagnosis code, sub-discharge diagnosis code, oxygen therapy (nasal cannula, high-flow nasal cannula, mechanical ventilator, and ECMO) within 2 weeks from the index date, surgical procedures, patient outcomes, and prescribed medications (such as corticosteroids [dexamethasone, methylprednisolone, prednisolone, and hydrocortisone], anticoagulants [heparin, dalteparin, and enoxaparin], antiplatelet agents, and vasopressors [norepinephrine, epinephrine, dopamine, and dobutamine]) within 2 weeks from the index date (Tables S1–S4).

### 2.4. Statistical Analysis

Categorical variables were expressed as frequencies and percentages, while continuous variables were expressed as medians and interquartile ranges. To compare the transfusion and non-transfusion groups, propensity score matching (1:2) was conducted based on sex and age. Subsequently, we compared and analyzed the risk factors for transfusion, including comorbidities; insurance type; medications; and procedures related to COVID-19 treatment, clinical severity, and treatment outcomes. Fisher’s exact test was used to assess the normality of categorical variables. Continuous variables were analyzed using the independent samples *t*-test for parametric methods and the Mann–Whitney U test for nonparametric methods, as appropriate.

The risk factors for blood transfusion in patients with severe COVID-19 were evaluated using a multivariate logistic regression analysis with the backward variable elimination method. The analysis utilized a balanced dataset, considering variables with a *p*-value < 0.001 as significant. The risk factors associated with 90-day mortality among these patients were assessed using multivariable Cox regression analysis to estimate the hazard ratio (HR) and the corresponding 95% confidence interval (CI). A *p*-value of <0.05 was considered significant. All statistical analyses were performed using SAS software (version 9.4; SAS Institute, Cary, NC, USA).

## 3. Results

### 3.1. Patient Selection and Blood Component Usage

During the study period, the NHIS-NHID identified 41,203 patients with severe COVID-19 who required oxygen therapy. Of these patients, 18,836 (45.7%) were ≥65 years, and 2941 (7.14%) received at least one type of blood product within 3 months of COVID-19 diagnosis (Table 1). A total of 41,385 units of blood products were transfused, including 14,477 units of pRBCs, 20,532 units of platelet concentrates, 4501 units of FFP, and 1875 units of cryoprecipitates (Table 1). Transfusion was significantly more common in patients aged ≥65 years than in individuals aged <65 years (*p* < 0.001), with 71.5% of all transfused patients falling into this age group. This trend was particularly pronounced in the transfusions of pRBCs and platelet concentrates (*p* < 0.001) but not observed in the transfusions of FFP (*p* = 0.115) and cryoprecipitates (*p* = 0.274).

Patients with higher clinical severity required larger amounts of blood transfusion. The amount of blood transfusion according to clinical severity was as follows. For pRBC transfusion, ECMO patients received an average of 13.29 units per patient, compared to 1.95, 0.44, and 0.92 units per patient for those on mechanical ventilation, high-flow nasal cannula, and simple oxygen inhalation, respectively. For platelet concentrate transfusion, ECMO patients received an average of 17.52 units per patient, compared to 3.13, 0.64, and 0.89 units per patient for those on mechanical ventilation, high-flow nasal cannula, and simple oxygen inhalation, respectively. For fresh frozen plasma transfusion, ECMO patients received an average of 4.23 units per patient, compared to 0.68, 0.16, and 0.18 units per patient for those on mechanical ventilation, high-flow nasal cannula, and simple oxygen inhalation, respectively.

### 3.2. Risk Factors Associated with the Transfusion of Blood Components

Propensity score matching analysis was performed to obtain a 1:2 matched sample of patients who received blood transfusions and those who did not by adjusting for baseline covariates, including age and sex. Of the 2941 individuals who underwent blood transfusion, 26 were excluded from the analysis due to their significantly different baseline propensity scores, indicating a high level of bias. Finally, 2915 individuals were included in the analysis of the transfusion group. Their average age was 70.74 ± 14.03 years, and 71.56% of the patients were ≥65 years. Men accounted for 53% of the total patients (Table 2). Patients in the transfusion group had a significantly higher prevalence of underlying diseases such as diabetes mellitus, cardiovascular disease, cerebrovascular disease, chronic kidney disease, and malignancy compared with the non-transfusion group, with a significantly higher Charlson Comorbidity Index (CCI) (Table 2).

The multivariate logistic regression analysis identified underlying chronic kidney diseases (OR: 1.73, 95% CI: 1.47–2.02), surgery (OR: 5.04, 95% CI: 4.419–5.749), thrombolysis or thrombectomy (OR: 2.21, 95% CI: 1.71–2.85), renal replacement therapy (OR: 5.04, 95% CI: 4.42–5.75), mechanical ventilation (OR: 1.25, 95% CI: 1.04–1.36), and ECMO (OR: 32.92, 95% CI, 19.37–55.96) as significant risk factors for blood transfusion in patients with severe COVID-19. Conversely, underlying cerebrovascular diseases (OR: 0.59, 95% CI: 0.40–0.87), a CCI score of ≥6 (OR: 0.67, 95% CI: 0.59–0.76), ICU admission (OR: 0.56, 95% CI: 0.50–0.63), and anticoagulant use (OR: 0.27, 95% CI: 0.24–0.30) were identified as protective factors (Table 3).

### 3.3. Treatment and Clinical Outcomes

Among the 8745 patients with severe COVID-19, no differences were observed in the frequency of corticosteroid prescriptions between the two groups. However, the usage rates of anticoagulant agents and life support measures, including mechanical ventilation and ECMO, were significantly higher in the transfused group compared to those in the non-transfused group (Table 2). Of the patients treated with ECMO, only 15 did not require blood transfusion. Additionally, renal replacement therapy, surgery, thrombolysis, and thrombectomy were more commonly performed in the transfusion group than in the non-transfusion group (Table 2). Notably, both the 30-day and 90-day mortality rates were significantly higher in the transfusion group (Table 2).

### 3.4. Risk Factors Associated with 90-Day Mortality

The multivariable Cox regression analysis identified underlying chronic kidney diseases (HR: 1.19, 95% CI: 1.02–1.39), a CCI score of ≥6 (HR: 1.71, 95% CI: 1.50–1.95), surgery (HR: 1.30, 95% CI: 1.14–1.48), renal replacement therapy (HR: 2.01, 95% CI: 1.62–2.49), mechanical ventilation (HR: 6.51, 95% CI: 5.67–7.46), ECMO (HR: 2.98, 95% CI: 3.16–5.02), and transfusion of blood components (HR: 1.36, 95% CI: 1.21–1.53) as the risk factors associated with 90-day mortality in patients with severe COVID-19. Conversely, ICU admission (HR: 0.79, 95% CI: 0.70–0.79), corticosteroid use (HR: 0.41, 95% CI: 0.33–0.41, *p* < 0.001), and anticoagulant use (HR: 0.66, 95% CI: 0.59–0.74, *p* < 0.001) were associated with a reduced risk of 90-day mortality (Table 4).

## 4. Discussion

We observed that 7.14% of the patients with severe COVID-19 requiring oxygen therapy received blood transfusions within 3 months of diagnosis in the ROK. Patients who required blood transfusions had a higher disease burden due to underlying conditions and clinical severity, and were more frequently subjected to invasive procedures compared to those who did not require transfusions. Notably, blood transfusion revealed a significant correlation with mortality rates, underscoring the necessity of effectively managing this resource in treating patients with severe COVID-19 during the pandemic.

The transfusion of blood components in patients with severe COVID-19 was associated with worse outcomes, consistent with findings from previous international and national studies [13,14,15,16,17]. The primary type of blood product used was pRBCs, corroborating our findings. In a prospective multinational study involving 28 European countries, pRBC transfusion was independently linked to HR of 1.67 (95% CI: 1.24–2.25) for 90-day mortality [13]. Similarly, an Italian multicenter study demonstrated that the number of pRBC units transfused was significantly associated with mortality in patients admitted to the ICUs [6]. Additionally, a Korean study using NHID data on COVID-19 demonstrated a positive association between blood transfusion and all-cause mortality [15].

Notably, our analysis revealed that blood transfusion served as an independent predictor for 30-day mortality, demonstrating a protective effect (HR: 0.60, 95% CI: 0.53–0.68) (Table S5). These findings suggest that blood transfusions may confer short-term benefits and long-term detrimental effects on the treatment outcomes in patients with COVID-19. The findings are consistent with previous reports involving patients undergoing cardiac surgery and acute coronary syndrome, which also indicated an association between blood transfusion and long-term mortality [18,19,20]. However, owing to the nature of this population-based study using claims data from the NHIS-NHID, detailed analyses of prescription patterns for blood products and their effects were not feasible. Further studies should evaluate the effect of restrictive transfusion strategies on mortality based on the criteria under which blood transfusion is deemed beneficial, particularly in critically ill, non-bleeding patients with COVID-19. This research will enhance our understanding of the pathophysiology of COVID-19 and aid in establishing a strategy for optimizing blood transfusion.

A notable finding of our study was the relatively large volume of blood products transfused compared to that of other studies. For instance, in a Brazilian study involving 3014 hospitalized patients, despite a similar severity profile—31% requiring mechanical ventilation—only 3187 units of blood products were transfused compared with 41,385 units in our study [14]. Although the transfusion criteria were not specified in the study, 72.4% of patients who received transfusion had an initial Hb ≤ 8.0 g/dL. Similarly, a United States (US) study reported an average transfusion volume of 4.8 units per patient with COVID-19, with 7.3% of patients on mechanical ventilators and approximately 70% of compliance with the restrictive transfusion guidelines; however, our study, with 25.6% of patients on mechanical ventilators, revealed a significantly higher overall transfusion volume of 14.1 units [21]. This discrepancy may be due to the differences in the availability of medical resources and clinical criteria for blood transfusions in each region. Furthermore, the variations in clinical severity and the frequency of bleeding played a significant role in influencing the number of blood transfusions.

From this perspective, the relatively large volume of blood products transfused in our cohort may be partly attributed to the high proportion of patients on ECMO (5.31%). Notably, ECMO was a significant predictor of blood transfusion in our study, with 96.8% of the ECMO group receiving blood transfusions. As is well-established, ECMO support can be associated with several complications, including bleeding and the need for blood product transfusion [11,22]. For example, a United Kingdom study reported that ECMO patients with COVID-19 received up to four times more blood transfusions than non-ECMO patients, while a US study reported that ECMO patients with COVID-19 required an average of 18.4 units of pRBC transfusion [11,23].

In our cohort, 67.55% of patients received anticoagulants, with higher usage in the transfusion group. Current guidelines for managing coagulopathy, a poor prognostic factor, recommend considering prophylactic doses of low-molecular-weight heparin for all patients with COVID-19 requiring hospital admission [24,25]. Anticoagulants may reduce the need for transfusions by preventing DIC, which can otherwise lead to increased bleeding and higher transfusion requirements. Our study demonstrated that anticoagulant use significantly reduced both the likelihood of blood transfusion and mortality. However, bleeding, mostly associated with anticoagulants, was a primary reason for pRBC transfusion in patients with COVID-19 during the early stages of the pandemic [10]. Future studies exploring the interplay among anticoagulation, bleeding events, and blood product use in these populations are warranted.

Unexpectedly, in our cohort, we identified underlying cerebrovascular diseases, a CCI score of ≥6, and ICU admission as protective factors against blood transfusion. The mechanism underlying these findings remains unclear; however, potential factors such as the preventive effects of medications taken for underlying diseases or the severity of patients admitted to the ICU may have influenced the outcomes. These results differ from those of the previous studies and require further investigation [14,15,26].

Our study has several limitations. First, the transfusion threshold was determined at the clinician’s discretion, likely varying across centers due to differing practice policies. Additionally, the possible inclusion of patients with do-not-resuscitate orders who were not actively transfused, despite medical indications, may have introduced confounding factors. Second, the use of NHIS data may have resulted in an overestimation of disease severity, as claims data were tied to insured health benefits and not directly linked to each hospital’s electronic medical records. This limitation might have led to incomplete capture of accurate diagnoses and prescriptions. Particularly, access to information such as specific transfusion indications, dosing regimens, or whether transfusions were administered prophylactically or therapeutically, was limited. Additionally, clinical data on laboratory findings, clinical context of active bleeding or coagulopathy, and the use of blood products as supportive measures in cases of anticoagulation-associated bleeding were unavailable for individual patients. Consequently, an optimized blood transfusion algorithm could not be derived from our findings. Lastly, the reliance on insurance claims data, which lacked detailed clinical information, posed a risk of error or case misclassification. Despite these limitations, the strength of this study lies in its inclusion of a large patient population and the use of comprehensive, nationwide electronic datasets.

## 5. Conclusions

This nationwide population-based study offers valuable insights into the prescription patterns of blood transfusions and associated risk factors in patients with severe COVID-19. Blood transfusion was independently associated with increased 90-day mortality, underscoring its association with poor outcomes in critically ill patients with COVID-19. These findings highlight the need to establish and validate transfusion strategies to improve treatment outcomes in this population. Additionally, whether patients with severe COVID-19 require more blood transfusions than those with other critical illnesses and the long-term effects of transfusions on patient outcomes warrant further investigation.

## Figures and Tables

**Table 1:** Blood component utilization in patients with severe COVID-19.

Age (yr)	Total	Red Blood Cells	Platelet Concentrate	Fresh Frozen Plasma	Cryoprecipitate
	Number of Patients				
<65	834 (3.7%)	760	323	230	51
≥65	2107 (11.2%)	1874	707	358	50
Total	2941 (7.1%)	2634	1030	588	101
**Age (yr)**	**Number of blood component units**				
<65	16,867	6301	7182	2169	1205
≥65	23,691	8146	12,543	2332	670
Total	41,385	14,477	20,532	4501	1875

**Table 2:** Comparison of demographic and clinical characteristics according to blood transfusion status in patients with severe COVID-19 after a 1:2 propensity score matching.

	Total (*n* = 8745)	Transfusion Group (*n* = 2915)	Non-Transfusion Group (*n* = 5830)	*p*-Value
Age (yr), mean ± SD	70.74 ± 14.03	70.74 ± 14.03	70.74 ± 14.03	
≥65 yrs, *n* (%)	6258 (71.56)	2086 (71.56)	4172 (71.56)	
Sex (male), *n* (%)	4635 (53)	1551 (53)	3090 (53)	
Comorbidities, *n* (%)				
Diabetes mellitus	6558 (74.99)	2353 (80.72)	4205 (72.13)	**<0.0001**
Cardiovascular diseases	3260 (37.28)	1180 (40.48)	2080 (35.68)	**<0.0001**
Cerebrovascular diseases	3402 (38.90)	1227 (42.09)	2175 (37.31)	**<0.0001**
Dementia	3373 (38.57)	1195 (40.99)	2178 (37.36)	0.0010
Chronic kidney diseases	1143 (13.07)	614 (21.06)	529 (9.07)	**<0.0001**
Malignancy	2398 (27.42)	984 (33.76)	1414 (24.25)	**<0.0001**
Charlson Comorbidity Index, median (IQR)	6 (4–8)	7 (5–9)	5 (7–9)	**<0.0001**
0–2	1236 (14.13)	277 (9.50)	959 (16.45)	**<0.0001**
3–5	3035 (34.71)	893 (30.63)	2142 (36.74)	**<0.0001**
≥6	4474 (51.16)	1745 (59.86)	2729 (46.81)	**<0.0001**
Treatment of severe COVID-19, *n* (%)				
Surgery	4004 (45.79)	2300 (78.90)	1704 (29.23)	**<0.0001**
ICU admission	5434 (62.14)	2242 (76.91)	3192 (54.75)	**<0.0001**
Corticosteroids	7758 (88.71)	2563 (87.92)	5195 (89.11)	0.0992
Anti-coagulants	5907 (67.55)	2163 (74.02)	3744 (64.22)	**<0.0001**
Anti-platelet agents	5926 (67.76)	2169 (74.41)	3757 (64.44)	**<0.0001**
Thrombolysis or thrombectomy	421 (4.81)	290 (9.95)	131 (2.25)	**<0.0001**
Oxygen therapy, *n* (%)				
Simple oxygen inhalation	2472 (28.27)	824 (28.27)	1648 (28.27)	1
High-flow nasal cannula	3568 (40.80)	559 (19.18)	3009 (51.61)	**<0.0001**
Mechanical ventilation	2241 (25.63)	1083 (37.15)	1158 (19.86)	**<0.0001**
Extracorporeal membrane oxygenation	464 (5.31)	449 (15.40)	15 (0.26)	**<0.0001**
Renal replacement therapy, *n* (%)	565 (6.46)	451 (15.47)	114 (1.96)	**<0.0001**
Clinical severity, *n* (%)				
Severe or critical cases	6273 (71.73)	2091 (71.73)	4182 (71.73)	1
30-day mortality	2303 (26.34)	912 (31.29)	1391 (23.86)	**<0.0001**
90-day mortality	3184 (36.41)	1567 (53.76)	1617 (27.74)	**<0.0001**
Complication, *n* (%)	4716 (53.93)	1746 (59.90)	2970 (50.9%)	**<0.0001**
Stroke	725 (8.29)	291 (9.98)	434 (7.44)	**<0.0001**
Acute myocardial infarction	118 (1.35)	48 (1.65)	70 (1.20)	0.0884
Thrombotic events	4339 (49.62)	1609 (55.20)	2730 (46.83)	**<0.0001**

Bold value indicates *p* < 0.05. ICU, intensive care unit; IQR, interquartile range.

**Table 3:** Multivariable analysis of risk factors associated with the transfusion of blood components in patients with severe COVID-19, based on the backward variable elimination method using the balanced dataset.

Variables	Odds Ratio	95% Confidence Interval	*p*-Value
Cardiovascular diseases	1.25	0.84–1.87	0.270
Cerebrovascular diseases	0.59	0.40–0.87	**0.008**
Chronic kidney diseases	1.73	1.47–2.02	**<0.001**
Malignancy	1.07	0.95–1.21	0.246
CCI score ≥ 6	0.67	0.59–0.76	**<0.001**
ICU admission	0.56	0.50–0.63	**<0.001**
Surgery	5.04	4.42–5.75	**<0.001**
Anticoagulants	0.27	0.24–0.30	**<0.001**
Thrombolysis or thrombectomy	2.21	1.71–2.85	**<0.001**
Renal replacement therapy	5.04	4.42–5.75	**<0.001**
Mechanical ventilation	1.25	1.04–1.36	**0.013**
ECMO	32.93	19.37–55.96	**<0.001**

Bold value indicates *p* < 0.05. CCI, Charlson Comorbidity Index; ECMO, extracorporeal membrane oxygenation; ICU, intensive care unit.

**Table 4:** Multivariable analysis of risk factors associated with 90-day mortality in patients with severe COVID-19, based on the backward variable elimination method using the balanced dataset.

Variables	Hazard Ratio	95% Confidence Interval	*p*-Value
Cardiovascular diseases	1.17	0.8–1.72	0.425
Cerebrovascular diseases	0.98	0.67–1.44	0.934
Chronic kidney diseases	1.19	1.02–1.39	**0.03**
Malignancy	1.00	0.89–1.13	0.959
CCI score ≥ 6	1.71	1.50–1.95	**<0.001**
ICU admission	0.79	0.70–0.79	**<0.001**
Surgery	1.30	1.14–1.48	**<0.001**
Transfusion	1.36	1.21–1.53	**<0.001**
Corticosteroids	0.41	0.33–0.41	**<0.001**
Anticoagulants	0.66	0.59–0.74	**<0.001**
Renal replacement therapy	2.01	1.62–2.49	**<0.001**
Mechanical ventilation	6.51	5.67–7.46	**<0.001**
ECMO	2.98	3.16–5.02	**<0.001**

Bold value indicates *p* < 0.05. CCI, Charlson Comorbidity Index; ECMO, extracorporeal membrane oxygenation; ICU, intensive care unit.

## Data Availability

Data supporting the findings of this study are available upon request from the corresponding authors.

## Supplementary Materials

The following supporting information can be downloaded at: https://www.mdpi.com/article/10.3390/jcm13237327/s1, Table S1: International Classification of Diseases, Tenth Revision codes used to determine blood products transfused; Table S2: International Classification of Diseases, Tenth Revision codes used to classify treatment and clinical outcomes; Table S3: Anatomical Therapeutic Chemical Classification System codes used for determining COVID-19-related treatment; Table S4: International Classification of Diseases, Tenth Revision codes used for determining the comorbidities based on the Charlson Comorbidity Index; Table S5: Multivariable Cox regression analysis of the 30-day mortality in patients with COVID-19.
