# Supplementary material for: Blood Transfusion Utilization in Patients with Severe Coronavirus Disease 2019 in the Republic of Korea: A Nationwide Population-Based Study
Source: J Clin Med. 2024 Dec 2;13(23):7327. doi: 10.3390/jcm13237327 (PMC11642700; doi:10.3390/jcm13237327)
Supplement: Supplementary file 1 [file jcm-13-07327-s001.zip › jcm-3260798-supplementary.pdf]

Supplementary Materials

Table S1. International Classification of Diseases, Tenth Revision codes used to determine blood products transfused

Blood products	Codes
**Red blood cell**	X2021, X2022, X2031, X2032, X2091, X2092, X2111, X2112, X2131, X2132, X2512, and X2515
**Platelet concentrate**	X2081, X2082, X2121, X2122, X2501, X2511, X2513, and X2516
**Fresh frozen plasma**	X2011, X2012, X2071, X2072, X2141, X2142, X2504, X2514, X2041, X2042, X2051, and X2052
**Cryoprecipitate**	X2061 and X2062

Table S2. International Classification of Diseases, Tenth Revision codes used to classify treatment and clinical outcomes

Treatment	Codes
**Simple Oxygen inhalation**	M0040
**High-flow nasal cannula**	M0046
**Mechanical ventilation**	M5850, M5857, M5858, M5859, and M5860
**ECMO**	O1901, O1902, O1903, and O1904
**Renal replacement therapy**	O7031, O7032, O7033, O7034, O7035, O7051, O7052, O7053, O7054, and O7055
**Surgery**	OPRTN_YN
**Thrombolysis or thrombectomy**	AP506, AP606, AP706, AP806, M6630, M6631, M6632, M6634, and M6635
**ICU admission**	CZ_ITEM_CD 0203

ICU, intensive care unit; ECMO, extracorporeal membrane oxygenation

Table S3. Anatomical Therapeutic Chemical Classification System codes used for determining COVID-19-related treatment

Medication	ATC code
**Corticosteroids**	
Dexamethasone	H02AB02
Methylprednisolone	H02AB04
Prednisolone	H02AB06
Hydrocortisone	H02AB09
**Anticoagulants**	
Heparin	B01AB01
Dalteparin	B01AB04
Enoxaparin	B01AB05

COVID-19, coronavirus disease 2019

Table S4. International Classification of Diseases, Tenth Revision codes used for determining the comorbidities based on the Charlson Comorbidity Index

Comorbidities	ICD-10 codes
Myocardial infarction	I21, I22, and I252
Congestive heart failure	I099, I110, I130, I132, I255, I420, I425, I426, I427, I428, I429, I43, I50, and P290
Peripheral vascular disease	I70, I71, I731, I738, I739, I771, I790, I792, K551, K558, K559, Z958, and Z959
Cerebrovascular disease	G45, G46, I60, I61, I62, I63, I64, I65, I66, I67, I68, I69, and H340
Dementia	F00, F01, F02, F03, G30, F051, and G311
Chronic pulmonary disease	I278, I279, J40, J41, J42, J43, J44, J45, J46, J47, J60, J61, J62, J63, J64, J65, J66, J67, J684, J701, and J703
Rheumatic disease	M05, M06, M315, M32, M33, M34, M351, M353, and M360
Peptic ulcer disease	K25, K26, K27, and K28
Mild liver disease	B18, K700, K701, K702, K703, K709, K713, K714, K715, K717, K73, K74, K760, K762, K763, K764, K768, K769, and Z944
Moderate or severe liver disease	I850, I859, I864, I982, K704, K711, K721, K729, K765, K766, and K767
Diabetes without complications	E100, E101, E106, E108, E109, E110, E111, E116, E118, E119, E120, E121, E126, E128, E129, E130, E131, E136, E138, E139, E140, E141, E146, E148, and E149
Diabetes with complications	E102, E103, E104, E105, E107, E112, E113, E114, E115, E117, E122, E123, E124, E125, E127, E132, E133, E134, E135, E137, E142, E143, E144, E145, and E147
Paraplegia and hemiplegia	G041, G114, G800, G81, G82, G830, G831, G832, G833, G834, and G839
Renal disease	I120, I131, N030, N031, N032, N033, N034, N035, N036, N037, N038, N039, N050, N051, N052, N053, N054, N055, N056, N057, N058, N059, N18, N19, N250, Z490, Z491, Z492, Z940, and Z992
Any malignancy	C00, C01, C02, C03, C04, C05, C06, C07, C08, C09, C10, C11, C12, C13, C14, C15, C16, C17, C18, C19, C20, C21, C22, C23, C24, C25, C26, C30, C31, C32, C33, C34, C37, C38, C39,

	C40, C41, C43, C45, C46, C47, C48, C49, C50, C51, C52, C53, C54, C55, C56, C57, C58, C60, C61, C62, C63, C64, C65, C66, C67, C68, C69, C70, C71, C72, C73, C74, C75, C76, C77, C78, C79, C80, C81, C82, C83, C84, C85, C88, C90, C91, C92, C93, C94, C95, C96, and C97
Organ transplantation	Z94

Table S5. Multivariable Cox regression analysis of the 30-day mortality in patients with COVID-19

Variables	Hazard ratio	95% confidence interval
Cardiovascular diseases	1.21	0.80–1.81
Cerebrovascular diseases	0.92	0.61–1.37
Chronic kidney diseases	1.37	1.17–1.6
Malignancy	0.92	0.82–1.05
Charlson Comorbidity Index ≥ 6	1.64	1.43–1.88
ICU admission	0.70	0.62–0.8
Surgery	1.17	1.01–1.35
Transfusion	0.62	0.54–0.7
Corticosteroids	0.34	0.30–0.39
Anticoagulants	0.60	0.53–0.68
Renal replacement therapy	2.26	1.84–2.77
Mechanical ventilation	6.80	5.88–7.87
ECMO	3.74	2.90–4.82

ECMO, extracorporeal membrane oxygenation; ICU, intensive care unit; IQR, interquartile range
